# Single Cell Cryo-Soft X-ray Tomography Shows That Each *Chlamydia Trachomatis* Inclusion Is a Unique Community of Bacteria

**DOI:** 10.3390/life11080842

**Published:** 2021-08-18

**Authors:** Patrick Phillips, James M. Parkhurst, Ilias Kounatidis, Chidinma Okolo, Thomas M. Fish, James H. Naismith, Martin A. Walsh, Maria Harkiolaki, Maud Dumoux

**Affiliations:** 1 Diamond Light Source, Harwell Science and Innovation Campus, Didcot OX11 0DE, UK; patrick.phillips@diamond.ac.uk (P.P.); james.parkhurst@rfi.ac.uk (J.M.P.); ilias.kounatidis@diamond.ac.uk (I.K.); chidinma.okolo@diamond.ac.uk (C.O.); thomas.fish@diamond.ac.uk (T.M.F.); martin.walsh@diamond.ac.uk (M.A.W.); maria.harkiolaki@diamond.ac.uk (M.H.); 2 Research Complex at Harwell, Harwell Science and Innovation Campus, Didcot OX11 0DE, UK; naismith@strubi.ox.ac.uk; 3 Division of Structural Biology Department, University of Oxford, Roosevelt Drive, Oxford OX3 7BN, UK; 4 The Rosalind Franklin Institute, Harwell Science and Innovation Campus, Fermi Road, Didcot OX11 0FA, UK

**Keywords:** *Chalmydia*, cryo-soft X ray tomography, community, single cell

## Abstract

*Chlamydiae* are strict intracellular pathogens residing within a specialised membrane-bound compartment called the inclusion. Therefore, each infected cell can, be considered as a single entity where bacteria form a community within the inclusion. It remains unclear as to how the population of bacteria within the inclusion influences individual bacterium. The life cycle of *Chlamydia* involves transitioning between the invasive elementary bodies (EBs) and replicative reticulate bodies (RBs). We have used cryo-soft X-ray tomography to observe individual inclusions, an approach that combines 40 nm spatial resolution and large volume imaging (up to 16 µm). Using semi-automated segmentation pipeline, we considered each inclusion as an individual bacterial niche. Within each inclusion, we identifyed and classified different forms of the bacteria and confirmed the recent finding that RBs have a variety of volumes (small, large and abnormal). We demonstrate that the proportions of these different RB forms depend on the bacterial concentration in the inclusion. We conclude that each inclusion operates as an autonomous community that influences the characteristics of individual bacteria within the inclusion.

## 1. Introduction

*Chlamydiae* are pathogenic to both humans and animals, with impacts on livestock [1] and wildlife [2]. In humans, *Chlamydiae* are the primary cause of acquired blindness worldwide [3] and are a leading cause of bacterial sexually transmitted disease in developed countries [4]. They are also responsible for atypical pneumonias [5] and are associated with Reiter’s syndromes [6] and cardiovascular diseases [7].

*Chlamydiae* are intracellular bacteria found in two morphologically distinct forms: the elementary body (EB) (the non-dividing infectious form) and the reticulate body (RB) (the dividing non-infectious form). These forms inter-convert within a compartment named the inclusion. Having entered the cell, EBs convert to RBs, which multiply. RBs then convert back to EBs, which then break out of the cell, ensuring onward propagation by infecting neighbouring cells [8]. Therefore, during the life cycle, the bacterial inclusion forms, matures, expands and ultimately exits the cell [9]. Since the end point of the intracellular growth of *Chlamydiae* is the formation of an inclusion containing a large number of newly formed EBs, there is a link between the size of the inclusion and the maturation of the bacterial population [10]. The EB and RB forms of *Chlamydiae* have very different sizes (volumes) [11]. Within each inclusion, the bacterial community must coordinate two processes, RB replication and RB conversion to EB [9]. When studying the *Chlamydiae* life cycle, applications of common analytical techniques are very difficult as *Chlamydiae* are genetically intractable [12], and probes such as DNA intercalants or other chemicals interfere with the host cell function [13,14].

There are two main tomography approaches in biology, electron-rays or soft X-rays, both of which are routinely performed at cryo-temperature to maintain sample integrity. Cryo-electron tomography (cryo-ET) can reach nanometer resolution and with sub-tomographic averaging atomic resolution [15,16] but requires samples thinner than around 150 nm [17]. Serial block face electron microscopy, although not projection tomography, is a powerful approach for visualising 3D volumes, which circumvents the thickness limitation by imaging the surface layer, removing it and imaging the newly exposed surface. This process is, however, quite slow and often requires chemical fixation and heavy metal staining of the cells, all of which generate artefacts. Cryogenic soft X-ray tomography (Cryo-SXT) can interrogate much thicker samples (up to 10 µm). Cryo-EM has aided in simplifying sample preparation and expanding the scope of the technique [18]. However, the nominal resolution is usually restricted to 35–50 nm [19]. With the modification of the zone plate (ZP), 25 nm resolution can be achieved but this limits the depth of focus to 1 µm [19].

A study by Lee et al. in 2018 using serial block face electron microscopy (sbf-EM) has shed insights into the evolution of a *Chlamydia* inclusion [20]. Sbf-TEM, although powerful, took an average of 25 h per region of interest and generated up to 500 sections, each requiring manual segmentation. Moreover, sbf-EM uses fixed resin-embedded samples, which are mechanically sectioned. Fixation induces ‘membrane blisters’ [21] and damaged organelles such as mitochondria, whilst dehydration can remove molecules and form vacuoles [22]. Sectioning can introduce sample compression, thickness variation and knife marks [23,24]. Cryogenic soft X-ray tomography (cryo-SXT) provides the means to bypass these sample preparation induced artefacts and to image frozen hydrated chlamydia infected cells in their near native state at the 10 s of nanometer resolution range.

Here, we report the novel deployment of cryo-SXT to study the organisation of the *Chlamydia* inclusion at the stage where both active RB division and transformation into the EB form occur. We have used a semi-automated segmentation approach using freely available software. The data (40 nm nominal resolution, depth of focus 10 µm) demonstrate that bacterial concentration is an important factor in the regulation of the reticulate body volume.

## 2. Results

### 2.1. Imaging Chlamydia Inclusion with Cryo-SXT: Advantages and Limitations

**Figure 1:**
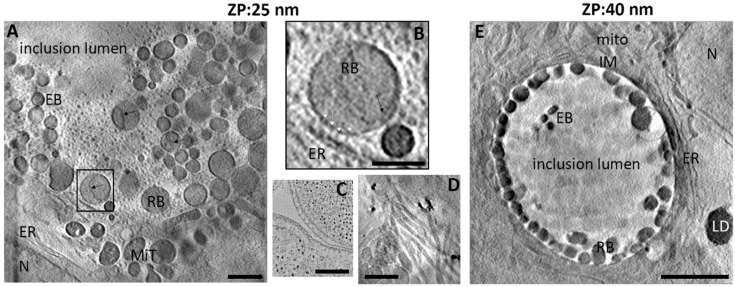
Observation using cryo-SXT and characterisation of HeLa cells infected by *C. trachomatis*. HeLa cells infected with C. trachomatis for 24 hpi and observed using cryo-SXT with a 25 nm zone plate (**A**,**B**,**D**) or 40 nm zone plate (**E**). MiT: mitochondria, N: nucleus, EB: elementary body, RB: reticulate, IM: inclusion membrane, ER: endoplasmic reticulum body, LD: lipid droplets black arrow: inner membrane invagination; (**B**) corresponds to the box in A and highlights the presence of the pathogen synapse with the T3SS array (white arrow heads); (**C**): from Dumoux et al. (2012), TEM of the pathogen synapse for reference. D: host cell cytoskeleton. Scale bars: 1 µm (**A**,**D**), 0.5 µm (**B**), 250 nm (**C**) and 5 µm (**E**).

HeLa cells infected with *C.trachomatis LGV2* were prepared on EM grids; the use of such 2D supports is standard in cellular imaging as it allows access for manipulation [25]. Finder grids were used to allow correlative microscopy to quickly locate the inclusion. We decided to work at low infectivity with inclusion forming units (IFU) between 0.5 and 0.8 to mimic physiological infection. Cryo-SXT data were acquired at Diamond Light Source on beamline B24 which can image at 25 nm or 40 nm resolution using absorption contrast imaging. At 25 nm, images showed *Chlamydia* inclusions at 24 hpi (hours post infection) with the expected features of *Chlamydia* and host cells (Figure 1A–D). The host cell cytoskeleton, mitochondrial cristae and the endoplasmic reticulum (ER) were clear (Figure 1A,B,D). The bacterial inner and outer membrane and the membrane invagination typical of the PVC (*Planctomycetes, Verrucomicrobia* and *Chlamydiae*) super-phylum [26] were visible (Figure 1A,B). We observed the characteristic accumulation of glycogen (grey amorphous globules) within the inclusion lumen [27] and the characteristic type III secretion system array in contact with the ER (the ‘pathogenic synapse’) (Figure 1B,C) [28]. This higher resolution cryo-SXT thus confirms that this approach yields images with the expected features of the system that are free from distortion. In order to image a volume sufficient for an entire inclusion in a single experiment, 40 nm resolution was used (Figure 1D, Supplementary Movies). At a lower 40 nm resolution, the inner and outer membranes were no longer distinct, and the glycogen granules were less defined. However, the inclusion membrane, the mitochondria, cytoskeleton and ER remained clearly visible. Crucially at this resolution, the entire inclusion was imaged in around 40 min (Figure 1E).

The tomographic reconstruction was performed using the IMOD software package [29]. This step consists of the alignment of the frames within the tilt series (a frame is an image at a certain angle) so that, after weighted back projection, a stack (series of z planes) can be obtained. The segmentation of the inclusion was completed using SuRVoS [30]. We then used ImageJ [31] to segment and measure the volume of individual bacteria within the inclusion (macro detailed in Supplementary Material). We relied on manual curation to remove the incorrect objects and add back objects which were incorrectly fragmented. This was simplified as each object was associated with an identification number. At the end of the process, every segmented object is associated with an identification number and a volume. The entire computational process, from tomogram reconstruction to segmentation including curation took approximatively 8 h per inclusion.

**Figure 2:**
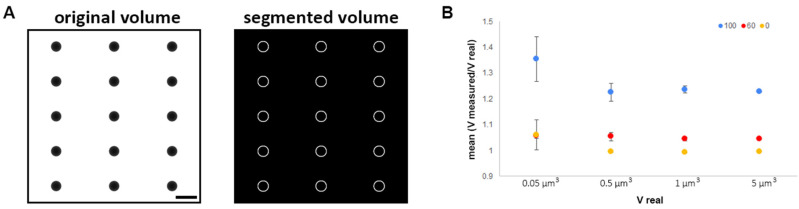
Impact of the missing wedge using simulation data composed of spheres of different volumes. (**A**) An example of segmented simulated data (0.5 µm^3^ spheres, 0° tilt). Raw data are on the left and the segmented volume, which recognises boundaries, on the right. Scale: 1 µm. (**B**) Mean ratio (measured volume/real volume) as a function of the real volume with different missing wedges of data (100° (blue) or 60° (red) or no missing wedge (yellow)).

**Figure 3:**
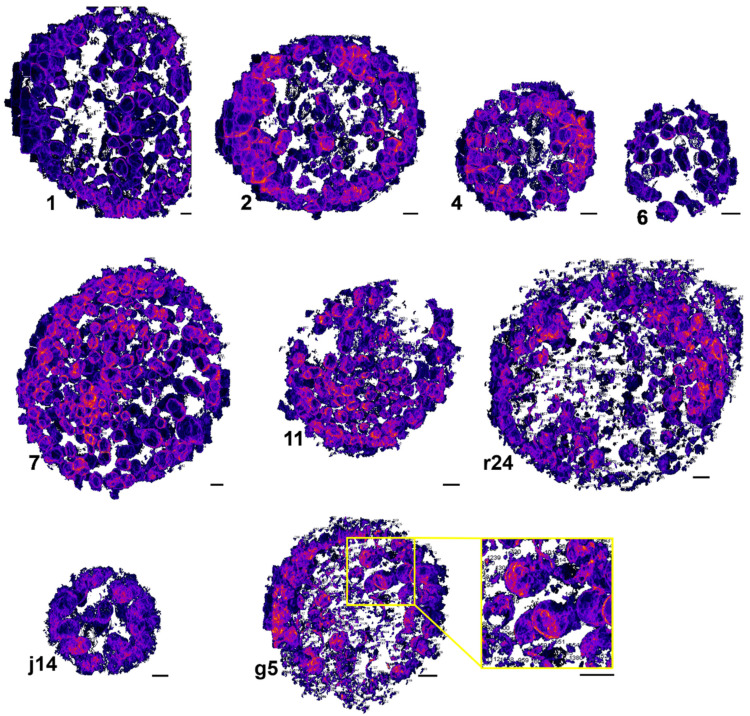
3D rendering of *C. trachomatis* inclusion at 24 hpi after using our proposed segmentation pipeline. Inclusions were isolated from the cells using SuRVoS and then bacteria were individually segmented using an ImageJ script. Presented in this is figure non-manually curated data after maximum intensity projection to reveal the volume information. Numbers are generated by ImageJ to identify every volume. In the yellow box is a zoomed in part of g5. Scale bar: 1 µm.

Projection tomography is based on the Radon transform [32] where a series of images of an object acquired uniformly over 180° can be transformed into Fourier space so that the object can be projected back to real space in its full volume. However, practical limitations, such as sample holder tilt range, sample thickness and sample carrier, often restrict the data acquisition to less than 180°. At B24 the setup allows projection images to be recorded from −70° to +70°. The consequence of an incomplete tilt range is referred to as ‘the missing wedge’ [33]. We simulated a set of tilt series containing several spheres (0.05, 0.5, 1 and 5 µm^3^) with different missing wedges (0°, 60° and 100°). The volumes were accurately calculated within 0.05% error when there was no missing wedge (Figure 2). As expected, the larger the missing wedge, the more inaccurate the volume calculations were. Smaller volumes were more severely affected than larger ones, this may also convolve segmentation errors. The presence of objects that obstruct the X-rays, such as grid bars, also remove data, and this is sample specific. Our approach was to record all tilt series with a uniform missing wedge of 85° so as to have a standardized approach relative to the unavoidable problem of the missing wedge of data. We used our segmentation pipeline to segment and calculate the volume of every individual bacterium (event) (Figure 3). 

In order to compensate for the missing wedge, the simulation of spheres of 0.08 µm^3^ and 0.3 µm^3^ representing the EB and RB, respectively [11], led us to derive a single correction factor for both sizes (alpha <0.01) (Figure S1). Hence, we were able to apply a correction factor to the measured size of the bacteria (which are intrinsically spherical) to correct for the missing wedge data.

### 2.2. Volume Calculation and Classification of Bacteria within the Chlamydia Inclusion

**Figure 4:**
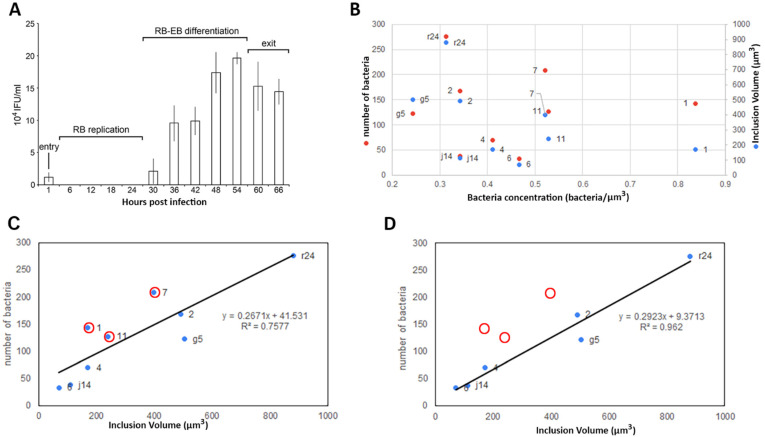
*C. trachomatis* inclusions at 24 hpi are widely different in volume and number of bacteria. (**A**) C. trachomatis titration assay in HeLa cells at MOI 0.8. HeLa cells have been infected with C. trachomatis and the cells have been collected at the different time points shown. Then, the pelleted infected cells have been used to infect a fresh layer of cells to quantify the inclusion forming units (IFUs). The phase of infection is determined with regards to the IFU. RB: reticulate bodies, EB: elementary body. (**B**) After segmentation, each inclusion (ID as a data label in the graph as a number or number plus letter) volume, number of bacteria and consequent bacteria concentration have been plotted. (**C**,**D**) Number of bacteria as a function of the inclusion volume ((**C**), full dataset; (**D**), partial dataset; red circle highlights the absence of inclusion 1, 11 and 7).

When considering a population of cells infected by *Chlamydia*, the infection is asynchronous as the size of an inclusion depends on the initial bacterial load which controls both maturation time and volume [10]. Typically, to establish an infection, a layer of cells at confluence between 70–90% was placed in the presence of a dilution of bacteria in their medium (Material and Methods). To promote synchronisation, a short (5 to 10 min) centrifugation at low speed (100 g) was used to concentrate bacteria at the cell surface. A population of infected cells was selected and judged to be reaching the same ‘phase’ of infection [34]; such a definition is inherently imprecise for individual cells. To identify which phase our sample were in, we performed an infectivity assay (Figure 4A). This assay uses a population of cells infected for the indicated time (in the x axis of Figure 4A) prior to collection and infection of a fresh layer of cells that would be left for 24 h before fixing, labelling and counting of the number of inclusion and cells. This allows the determination of the inclusion forming unit (IFU). In our samples, the inclusions were mostly formed of RBs (non-infectious) with some undergoing transition to EBs (Figure 4A). This is a complex phase of the life cycle, and the signal for the transition is unknown [9]. 

The number of bacteria and the volume of the inclusion were correlated (R^2^ = 0.7577) (Figure 4B,C), indicating bacterial concentration in the inclusion is a constant which is in agreement with Lee et al. (2018). However, three inclusions (7, 11 and 1) deviated from this correlation with higher numbers of bacteria per unit volume, removing these three inclusions from the analysis increased the correlation coefficient for the remaining inclusions (g5, r24, j14, 2, 4 and 6) to 0.962 (Figure 4D). Based on these data, we categorised the inclusions into two broad groups: ‘standard’ (g5, r24, j14, 2, 4 and 6) and ‘high concentration’ inclusions (7, 11 and 1).

**Figure 5:**
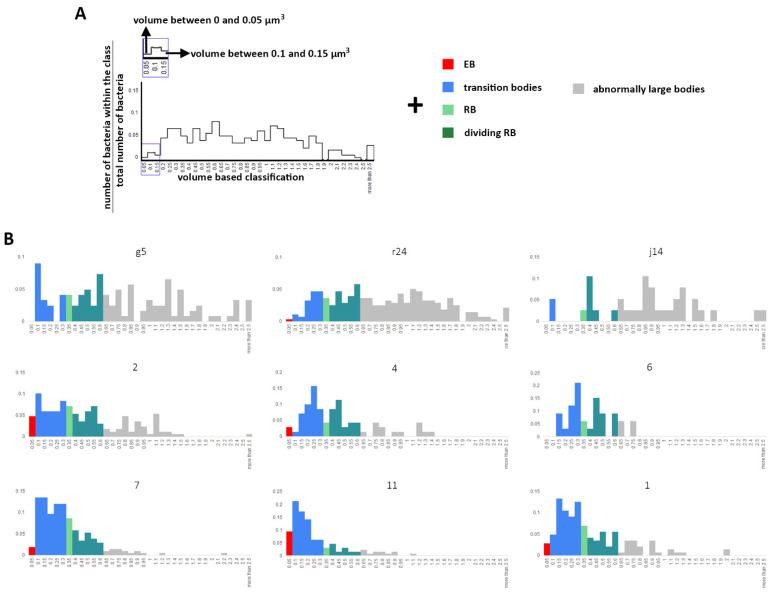
Classification of bacteria population using the most accepted literature. After segmentation, the volume of individual bacteria has been determined and grouped in volume classes (x-axis) and the ratio of bacteria within the class (y-axis). Then, the volume classes are grouped into higher classes, depicted by a colour and representing a bacteria form based on the most accepted literature. An example is presented in (**A**), and the whole dataset is present in (**B**). The Axis in (**B**) are as for (**A**). EB: elementary bodies, RB: reticulate bodies.

**Figure 6:**
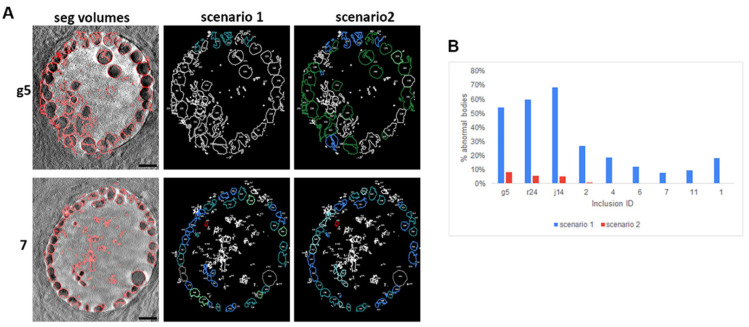
Over-representation of abnormally large body in scenario 1 (Figure 5) when compared to scenario 2 (Figure 7)**.** (**A**) Inclusions (7 and g5) were segmented with the same parameters following scenario 1 (presented in Figure 5) or 2 (presented in Figure 7), where volumes are associated with the most used in the literature or following Lee et al., respectively. Panels ‘seg volumes’ (segmented volumes); red highlights the boundary found by our segmentation pipeline. ‘Scenario 1′ and ‘scenario 2′ associate the delimited bacteria with its classification following higher classes and consequent colour coding as per Figure 5 and Figure 7, respectively. Scale bar: 5 µm. (**B**) Number of abnormal bodies per inclusion depending on the parameters used for classification (scenario 1 blue or 2 red).

Within inclusions, individual bacteria volumes were grouped by increments of 0.05 µm^3^. The long-standing literature model for *Chlamydia* has EB to be of the order of 0.3 µm in diameter, RB to be ~1 µm in diameter, persistent bodies (PB) or aberrant bodies (AB) to be ~2 µm in diameter and a series of transition bodies (from EB to RB and back; dividing RB; from AB to RB) [9,11,35]. We classified cells by their volume as ‘RB’, ‘EB’, ‘dividing RB’ and ‘transition bodies’ by using volumes derived from the diameters presented in this body of literature. Transition bodies are bacteria that are difficult to associate to a category without a time-resolved approach as we do not know at which stage and in which direction they are converting to. The volume of dividing RB was calculated by assuming two connected spheres. This method of assignment does not reflect the diversity of the bacterial volumes we observed. For example, in inclusion j14, 68% fell outside this classification and, therefore, have been assigned as abnormally large bodies (Figure 5 and Figure 6). 

**Figure 7:**
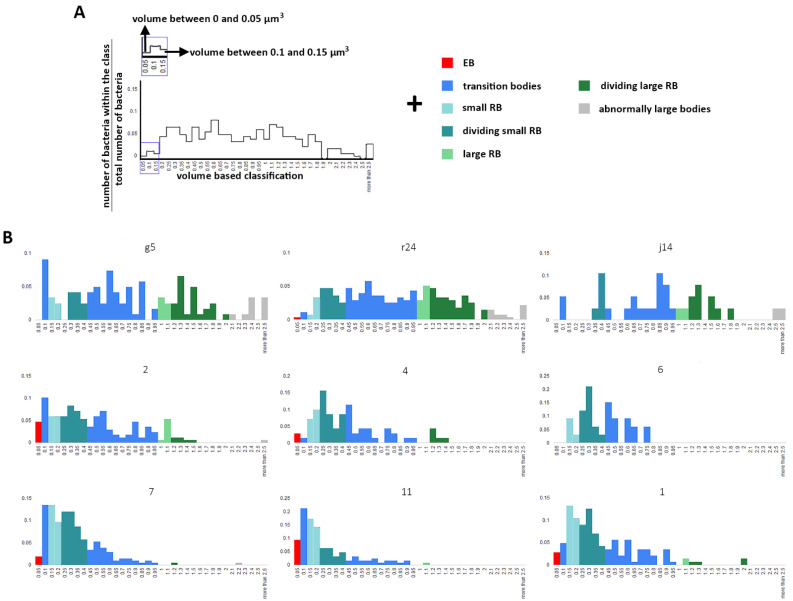
Classification of bacteria population using Lee et al. After segmentation, the volume of individual bacteria has been determined and grouped in volume classes (x-axis) and the ratio of bacteria within the class (y-axis). The volume classes are then grouped into cell classes depicted by a colour and representing a bacteria form based on Lee at al. An example is presented in (**A**) and the whole dataset is present in (**B**). The Axis in (**B**) are as for (**A**). EB: elementary bodies, RB: reticulate bodies.

Lee et al. in 2018 proposed a more complex classification where RB size varied over time from 0.8 µm^3^ at 16 hpi and 0.4 µm^3^ at 24 hpi to 0.2 µm^3^ at 32 hpi. The high standard deviation at 24 hpi and 32 hpi (100%) indicated that a range was present. Lee et al. reported diameters of 1.25 µm and 0.67 µm for the large and small RB. We used these diameters to derive a volume classification of our data (bacteria being considered as spheres). We reclassified bacteria (scenario 2, Figure 7) using the parameters of Lee et al. 2018 which added the classes: ‘small RB’ and ‘large RB’. This in turn added further classes ‘dividing small RBs’ and ‘dividing large RB’ (Figure 7, Methods), The population in blue represents the transition bodies present between the EB and RB and large and small RB, which are bacteria transitioning between the different forms. More than at least 92% of the bacterial population was assigned for inclusion g5 (Figure 6 and Figure 7); the analysis shows that different dividing forms co-exist, producing a variety of bacterial volumes. 

### 2.3. Bacteria Concentration Is Not Correlated with Transition to EB but Does Correlate with the RB Volume

**Figure 8:**
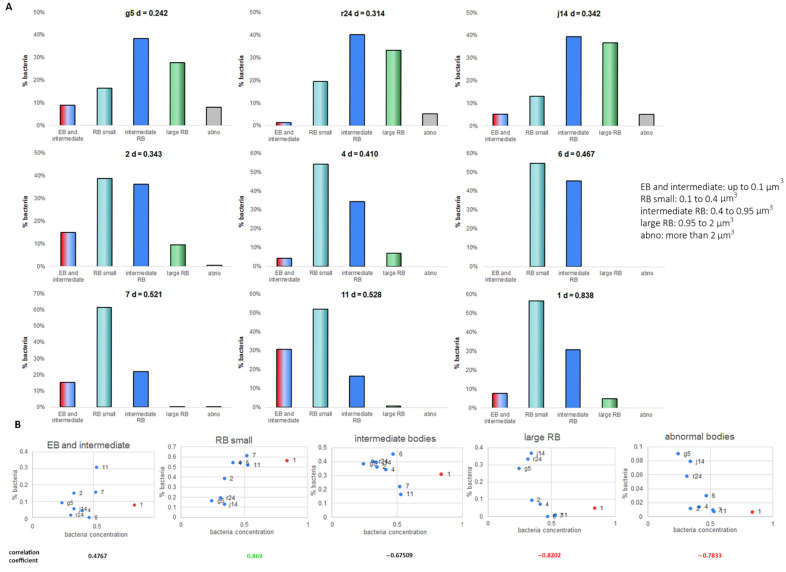
Bacteria classes vary depending on the concentration of bacteria inside the inclusion. (**A**) To ease the analysis, the main classes have been grouped to present 4 main classes. EB: elementary body; RB: reticulate body; d: density. (**B**) Proportion of bacteria as function of the concentration of bacteria (bacteria per µm^3^). In red, inclusion 1 which is atypical. Pearson’s correlation coefficient (without inclusion 1): in black under arbitrary threshold of ±0.75, in green positive correlation above 0.75 and in red negative correlation below −0.75.

Except for inclusion 11, which had 30% of EB or transitioning bacteria, EBs constituted between 0 and 25% of the total bacterial population. There was no strong correlation between the proportion of EB and the concentration of bacteria (number per volume) in the inclusions at 24 hpi (Figure 8A,B). However, inclusions with the lowest cell concentration at 24 hpi showed an accumulation of large RB and abnormal bodies (Figure 8A,B). As the cellular concentration increased, irrespective of the total number of bacteria (Figure 4, Figure 5 and Figure 7), the proportion of abnormally large bodies declined rapidly to zero, the proportion of the large RBs population decreased and the proportion of small RBs increased (Figure 8A,B). Where bacteria were at the point of transition between RB replication and the RB to EB differentiation (Figure 4A), we did not observe any correlation for the transition to EB with either the volume or density of the inclusion. However, bacteria concentration showed a positive correlation with the proportion of small RBs (0.869) and a negative correlation with the proportion of large RBs (R^2^ = −0.8202) and abnormal bodies (R^2^ = −0.7833) (Figure 8B) again with the exemption of inclusion 1, which is a very atypical inclusion (Figure 4B).

## 3. Discussion

Cryo-SXT is a powerful technique allowing the extraction of meaningful metrics from only a few tomograms when the object of interest is present in multiple copies. Reducing the missing wedge of data is highly desirable, but new approaches are needed to sample presentation, such as using cylindrical sample holders which have been implemented at the National Center for X-ray Tomography [19]. However, growing adherent cells and experiments such as transfection, infection or incubation with probes remains challenging using such an approach [33]. Even once data have been collected, segmentation remains a time-consuming bottleneck for data analysis; hence, we have developed a new workflow to improve the efficiency of segmentation. The ability to collect and analyse datasets in a faster and more automated manner, without resorting to population averages, will allow more complex problems to be tackled. Artificial intelligence (AI) and projects based on deep-learning (often with the help of the public) are key for scientists to entirely extract all available information from their samples and datasets [36]. 

In order to quickly acquire volume images while working with cryo-preserved thick samples, we used cryo-STX by using a 40 nm ZP. We were able to image, segment and characterise bacterial populations within individual *Chlamydia* intracellular inclusions. Imaging with cryo-SXT allows rapid acquisition of a tilt series (completed in 40 min per area of interest); reconstruction and segmentation takes less than 6–8 h per tomogram. The open-source workflow for segmentation will allow further improvements such as machine learning to be incorporated. Since the technique uses intact cells, it does not require fixation or staining and works at speed so it can be combined with live cell microscopy to observe the evolution of *Chlamydia*. The approach does suffer from distortions introduced due to the missing wedge. *Chlamydia* are mostly spherical objects allowing one to derive a correction factor using simulated data so that the volume calculations are not distorted by the missing wedge data issue. We have shown that cryo-SXT allows single inclusion imaging, which represents important additional functional information. This is because although there is a coordinated community within each inclusion, each individual inclusion develops asynchronously within the cell. Most approaches for imaging *Chlamydia* have to rely on global averages of infected cells, which masks diversity in the individual bacterial communities. One striking example of such variation is the size of the inclusion. In our dataset, the average size is 337.5 µm^3^ with a standard deviation of 259 µm^3^. The origin of such difference has yet to be determined, and is probably multi-factorial (including mechanical constrain, initial bacterial load and access to nutrients) [9].

Our imaging data suggest that the proportion of EB within an inclusion is not correlated with the bacterial concentration inside the inclusion. This suggests that the initial signal to transition from RB to EB is not a simple nutrient deficiency-based signaling effect but is more consistent with the hypothesis that the RB to EB transition is a multi-factorial event [37]. However, our dataset mainly reports on inclusions where the transition to EBs is nascent, and more data at different stages are required to pinpoint these parameters and their correlations.

The long-standing literature model for *Chlamydia* has EB of 0.3 µm in diameter, RB of 1 µm in diameter, persistent bodies (PB) or abnormal bodies (AB) of 2 µm in diameter and a series of transition bodies (from EB to RB and back, dividing RB, from AB to RB) [9]. This model failed to represent the diversity in bacteria we observed. This was in agreement with the conclusions of Lee et al. [20], who demonstrated that the RB population is more diverse with a range of volumes using sbf-EM. Lee et al. proposed that the volume of RB was a function of time: as cells aged, they became smaller. By applying the Lee classification of different RB sizes, we were largely able to fit the RB diversity we observed at 24 hpi. However, we observed that the proportion of the different RB sizes also varied between inclusions, which were all formed at the same time. This would argue against time dependence being the sole factor that determined RB size. Our data show that the RB size was correlated with the bacterial concentration (number of bacteria in an inclusion divided by the inclusion volume) within an individual inclusion. Inclusions with low bacterial concentrations showed high proportions of the large RB and a greater number of the even larger abnormal bodies. High bacterial concentration inclusions had a higher proportion of smaller RBs with no or only very few large abnormal bodies. Importantly, inclusions tend to show an increase in the bacterial concentration over time, which may account for the observation by Lee et al. [20] Engstrom et al. [9] demonstrated that the increase in inclusion volume does not require bacterial replication. They observed that inclusions with low bacteria cell concentration accumulated abnormally large bodies, consistent with what we now report.

During sbf-EM, the resolution allows the observation of different factors such as the presence of a nucleoid which is characteristic of EBs, allowing for finer and more accurate classification. However, to image large (areas and depth) volumes without sectioning, we chose a compromise resolution of 40 nm and, as a result, lost some of the fine structural data. We therefore classified the bodies present between the RBs and the EBs as transition bodies, the majority of which are found between large and small RBs. Further classification will require time-resolved techniques as well as characterisation of the protein expression profile. Determining how and when a larger/smaller body is formed and how the different RBs transition is an important future question.

Our work has established that cryo-SXT is a useful and very rapid technique for studying *Chlamydia*, an important bacterial pathogen. The results we have obtained with this new approach are consistent with more laborious approaches. By being able to focus on individual inclusions within the cell, our data suggest that the concentration of *Chlamydia* RB within the inclusion is a very important parameter in determining the range of bacterial forms. Here, we demonstrate that each inclusion presents a different profile of RB corresponding to a unique set of factors such as the local bacterial concentration. Future studies along the time domain using this approach will shed light on the evolution of the infection and community sensing [38], which may open new therapeutic opportunities. 

## 4. Material and Methods

### 4.1. Sample Preparation

HeLa cells were grown on 200 F1 grids R2/2 (Quantifoil) with one grid per chamber of an 8 well chamber slide (Ibidi, Gräfelfing, Germany). HeLa cells were infected with *C. trachomatis* LGV2 [28]. At 23 hpi, grids were mapped on an Evos FL2 using a 20x objective. At 24 hpi, grids were collected and blotted before the addition of 4 µL of fiducials (250 nm silver from Ursa Biosciences) and plunged frozen after a 1 sec back blotting (Leica EM GP), Wetzlar, Germany. 

### 4.2. Soft X-ray Tomography

Tilt series were collected on the UltraXRM-S/L220c X-ray microscope (Zeiss, previously Xradia, Oberkochen, Germany) at beamline B24, Diamond light source with a Pixis 1024 B CCD camera (Princeton instruments, Trenton, NJ, USA) and a 25 or 40 nm zone plate with X-rays of 500 eV. Tilt series were typically collected with an increment of 0.5°.

### 4.3. Tomogram Reconstruction

Raw X-ray tomograms were cropped in order to ensure they had the same tilt ranges and, thus, the same missing wedge angles. Then, tomograms were reconstructed using eTomo, which is part of the IMOD package [29] (https://bio3d.colorado.edu/imod//, accessed on 1 January 2020). The volumes were not filtered before entering the segmentation pipeline. Nonlinear Anisotropic Diffusion (NAD) filtering was used for display purposes for Figure 1. Movies present data binned by 2 for all axis.

### 4.4. Simulation Data

The simulations were performed at an energy of 500 eV matching the energy used in the experiments at B24 by using the attenuation length for Carbon of 331 nm at 500 eV (henke.lbl.gov/optical_constants/atten2.html, accessed on 1 December 2020) and using a pixel size of 16 nm mimicking the 40 nm ZP acquisition parameters. For each pixel, i, in the simulated image, the effective thickness of the sample, d_i_, was calculated assuming a parallel beam, and the intensity for each pixel was then calculated simply as I_i_ = I_0_ *exp*(−d_i_/l). In order to avoid pixel artefacts for small spheres sizes, each pixel was sampled in quadrants and then averaged.

### 4.5. Segmentation and Classification

For experimental tomograms, a first segmentation was completed by using SuRVoS [30] (https://diamondlightsource.github.io/SuRVoS/docs/installation, accessed on 1 Januay 2020) to generate a mask to isolate the inclusion from the host cells. This mask was exported and used in ImageJ [31] (https://imagej.nih.gov/ij/docs/install/windows.html, accessed on 1 Januray 2020) to limit the segmentation process to within the inclusion. Simulated data enters the segmentation pipeline at this point. Next, each 2D image was segmented, and the volume of each object in pixels cubed was generated using the 3D image counter plugin [39], which also provides a unique identification number for each reconstructed object. The data were then manually curated (Supplementary Figure S1). The volume of the inclusion was provided by SuRVoS in pixels cubed. All voxels were then converted to micrometre cubed and a correction factor was applied to correct for the missing wedges. Volumes were then classified using 0.05 µm^3^ increments. These are then grouped to form higher classes representing the different form of bacteria forms using the volume mostly found within the literature [9,11,35] or following Lee et al. [20]. In order to identify the range of volumes occupied by the bacteria during binary fission, the bacteria were approximated to be perfect spheres that are overlapping, and that the minimum distance that could separate them is the resolution limit (40 nm), while the maximum distance is the radius minus the same resolution limit.

The correlation coefficient has been calculated as follows.

$$Correl\;\left(X,Y\right)=\frac{\sum^{}\left(x-\bar{x}\right)\left(y-\bar{y}\right)}{\sqrt{\sum^{}{\left(x-\bar{x}\right)}^{2}\sum^{}{\left(y-\bar{y}\right)}^{2}}}$$

## Supplementary Materials

The following are available online at https://www.mdpi.com/article/10.3390/life11080842/s1, Figure S1: Statistics of the simulation data representing the EB and RB, Figure S2: Super Region Volume Segmentation (SuRVoS) to segment the inclusion from the cytosol, Figure S3: Combination of the SuRVoS annotated inclusion and its tomogram, Figure S4: Output of the segmentation and volume identification using imageJ macro, Figure S5: Output of the macro: composite of the segmented objects and the original tomogram, Video S1: title.The acquired tilt series were reconstructed using eTomo, with a final bin of 2 for x, y and z. Movie title include the reference ID of the inclusion (movie_1, movie_g5…).
