# Supplementary material for: Single Cell Cryo-Soft X-ray Tomography Shows That Each Chlamydia Trachomatis Inclusion Is a Unique Community of Bacteria
Source: Life (Basel). 2021 Aug 18;11(8):842. doi: 10.3390/life11080842 (PMC8399160; doi:10.3390/life11080842)
Supplement: Supplementary file 1 [file life-11-00842-s001.zip › Suppl Mat-revised.pdf]

Single cell cryo-soft X-ray tomography reveals the importance of bacteria concentration in the regulation of *Chlamydia* size

Patrick Phillips[1,2,4], James M. Parkhurst[1,3], Ilias Kounatidis[1], Chidinma Okolo[1], Thomas M. Fish[1], James H. Naismith[2,3], Martin A. Walsh[1,2], Maria Harkiolaki[1,2], Maud Dumoux[1,2,3*]

[1] Diamond Light Source, Harwell Science and Innovation Campus, Didcot, OX11 0DE, UK

[2] Research Complex at Harwell, Harwell Science and Innovation Campus, Didcot, OX11, UK

[3] the Rosalind Franklin Institute, Fermi Road, Didcot, OX11 0FA

[4]University of Oxford, Division of Structural biology department, Roosevelt Drive, Oxford, OX3 7BN

Supplementary figure:

	EB			RB						
	measured volume	measured/ real		measured volume	measured /real	F test				
	0.0091054	1.138176		0.3403039	1.134346		2.43E-16	df	14	
	0.0091054	1.138176		0.340992	1.13664				14	
	0.0091054	1.138176		0.3403039	1.134346					
	0.0091054	1.138176		0.340992	1.13664					
	0.0091054	1.138176		0.3403039	1.134346	T test	0.000513			
	0.0099697	1.246208		0.340992	1.13664					
	0.0099697	1.246208		0.3403039	1.134346					
	0.0099697	1.246208		0.340992	1.13664					
	0.0099697	1.246208		0.3403039	1.134346		**null hypoyhesis accepted**			
	0.0099697	1.246208		0.340992	1.13664		**applied average correction factor**			
	0.0093512	1.168896		0.3417375	1.139125			**1.160565**		
	0.0093512	1.168896		0.3417375	1.139125					
	0.0093512	1.168896		0.3417375	1.139125					
	0.0093512	1.168896		0.3417375	1.139125					
	0.0093512	1.168896		0.3417375	1.139125					
**average**		1.1844267			1.136704					
**stdev**		0.0470458			0.00202					

Supplementary Figure S1: Statistics of the simulation data representing the EB and RB.

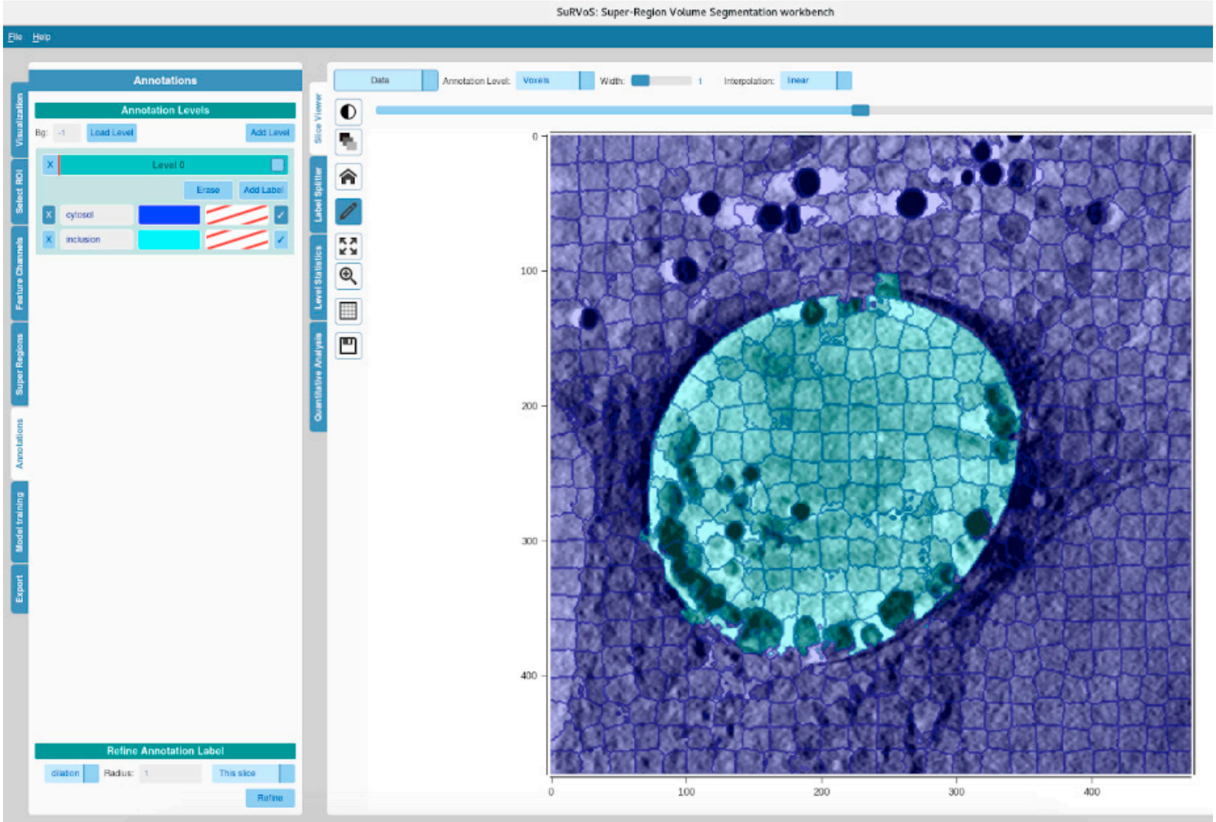

Supplementary Figure S2: Super Region Volume Segmentation (SuRVoS) to segment the inclusion from the cytosol. The dark blue grid depicts the calculated supervoxels which are optimised by the user to detect the edge of the inclusion. Some of the supervoxels are then manually annotated to label the inclusion and the cytosol. Then, using the model training tab, the program predicts further annotations based on the user's annotations to begin to automatically segment the inclusion from the cytosol throughout the entire tomogram.

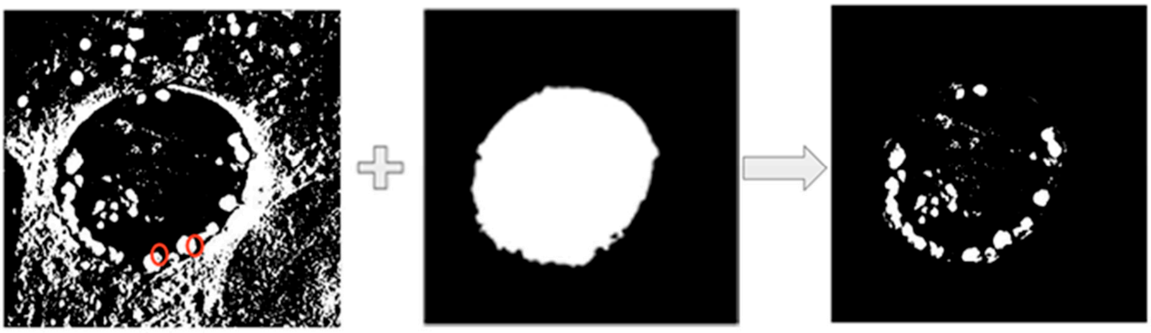

Supplementary Figure S3: Combination of the SuRVoS annotated inclusion and its tomogram. The tomogram is made binary and holes within the bacteria are filled (red circles). Then the SuRVoS annotation is added on top to black out the cytosol such that only the bacteria are counted and so that densities from the inclusion membrane do not contribute to the volume of the bacteria.

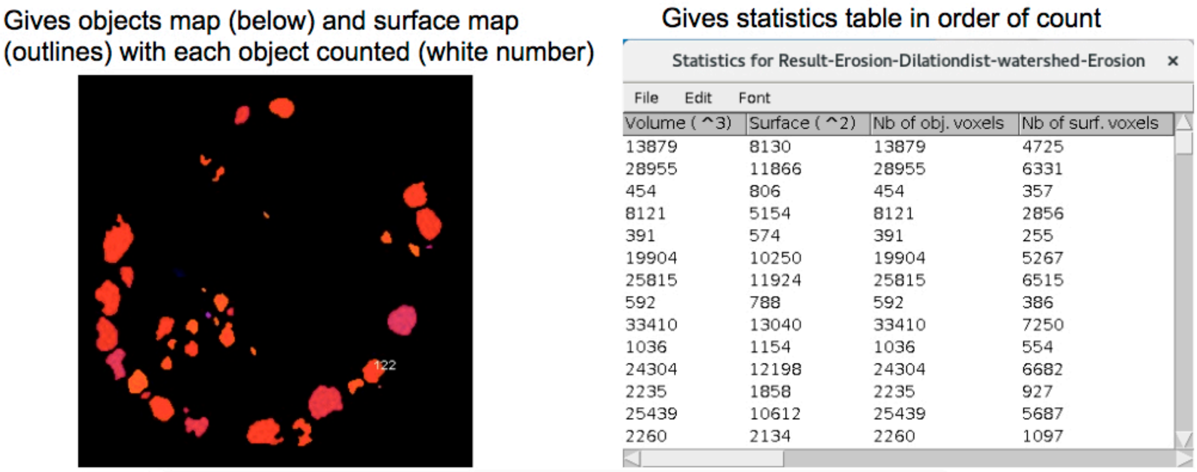

Volume (^3)	Surface (^2)	Nb of obj. voxels	Nb of surf. voxels
13879	8130	13879	4725
28955	11866	28955	6331
454	806	454	357
8121	5154	8121	2856
391	574	391	255
19904	10250	19904	5267
25815	11924	25815	6515
592	788	592	386
33410	13040	33410	7250
1036	1154	1036	554
24304	12198	24304	6682
2235	1858	2235	927
25439	10612	25439	5687
2260	2134	2260	1097

Supplementary Figure S4: Output of the segmentation and volume identification using imageJ macro. After running isolating the inclusion from the rest of the cells (Sup Fig 3) the 3D-objects-counter labels the centre of each object with a white number. The statistics table with the volumes of each object is given in the order of the counted objects (object 1 is the first in the list).

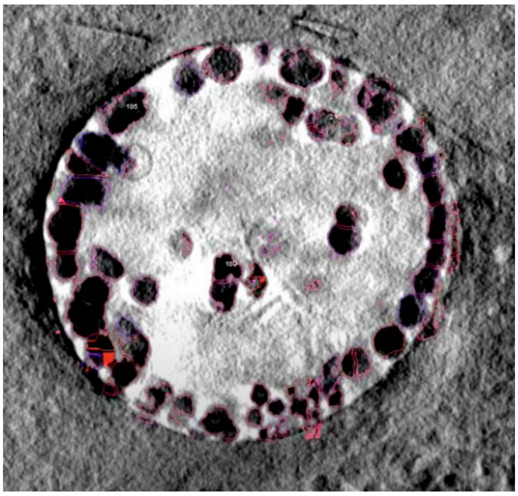

Supplementary Figure S5: Output of the macro: composite of the segmented objects and the original tomogram. Merging of the surface map of objects output by the 3D-objects-counter Each object is outlined and correspond to a unique identification number allowing manual curation if necessary. Each objects is labelled with a white number which is the count.

KEY RESOURCES TABLE

REAGENT or RESOURCE	SOURCE	IDENTIFIER
Antibodies		
Chlamydia trachomatis LGV2	ATCC	VR-902B

Bacterial and Virus Strains		
Chlamydia trachomatis LGVII	ATCC	VR-902B
Biological Samples		
HeLa	ATCC	CCL-2
Experimental Models: Cell Lines		
HeLa	ATCC	CCL-2
Software and Algorithms		
survos	diamond light source	https://diamondlightsource.github.io/SuRVoS/docs/installation/
image j	NIH	https://imagej.nih.gov/ij/
Other		

Supplementary information:

```
//Click Run and a window will open for you to select your tomogram, select the raw data tiff exported from SuRVoS

filepath = File.openDialog("Select tomogram");
open(filepath);
filename = File.getName(filepath);

// // Create dialog box - A dialogue box will pop up where you can select to enhance the contrast on your data

   //Dialog.create("Enhance Contrast?");
   //Dialog.addCheckbox("Run enhance contrast?", false);

   // Get dialog inputs
         //runEnhancecontrast = Dialog.getCheckbox();

   //if (runEnhancecontrast == true) {
         //run("Enhance Contrast...", "saturated=50 normalize process_all"); //enhance contrast on the data if ticked checkbox at start
   //}

//selectWindow("data.tif");
//setThreshold(0, 150);
run("Convert to Mask", "method=Default background=Light calculate black"); //the tomogram will be made binary

//A window will open for you to select your survos level 0 annotation .tif file
```

```
filepath = File.openDialog("Select survos level 0 annotation");
open(filepath);
annotation = File.getName(filepath);

//Create dialog box - A dialogue box will pop up where you can select parameters
   // select the radius you would like for the 'Closing' morphological filter on the SuRVoS
annotation
         //this will smooth the edge of the annotation and fill gaps
   // select the radius you would like for the morphological filters on the objects
         //this is the amount of erosion and dilation that will be used to smooth your objects
   // select the dynamic for the Distance Transform Watershed 3D
         //a lower dynamic will give more separations
   // select the radius you would like for the ersoion of the watershed image
         //the watershed image must be slightly eroded so that the 3D Objects Counter counts
touching objects (default 1)
   // select the circularity range
         //here you can filter out objects that are not circular (e.g.speckle), 1 is a perfect circle
   // choose to run a final despeckle if there is shadow and noise on the tomogram
         //this prevents background intensities contributing to the volume of your objects
   // select the size range of objects you want the 3D Objects Counter to count
         //this will not count objects that are too small or too large to be bacteria

Dialog.create("Choose parameters");
Dialog.addNumber("Radius for Closing Filter on the SuRVoS annotation (pixels):", 2);
Dialog.addCheckbox("Fill holes on SuRVoS annotation?", false);
Dialog.addNumber("Radius for Erosion and Dilation of the objects (pixels):", 1);
Dialog.addNumber("Dynamic for Distance Transform Watershed 3D (Lower=more
separations):", 1);
Dialog.addNumber("Radius for Erosion of the watershed (pixels):", 1);
Dialog.addNumber("Circularity min/max: ",  0.30);
Dialog.addToSameRow();
Dialog.addNumber("", 1.00);
Dialog.addCheckbox("Run final despeckle?", true);
Dialog.addNumber("Size min/max: ", 250);
Dialog.addToSameRow();
Dialog.addNumber("", 50000);
Dialog.show();

// Get dialog inputs
radiusValue = Dialog.getNumber();
runFillholes = Dialog.getCheckbox();
radiusValue = Dialog.getNumber();
dynamicValue = Dialog.getNumber();
```

```
radiusValue = Dialog.getNumber();
circularityMin = Dialog.getNumber();
circularityMax = Dialog.getNumber();
runDespeckle = Dialog.getCheckbox();
sizeMin = Dialog.getNumber();
sizeMax = Dialog.getNumber();

run("Make Binary", "method=Default background=Default calculate black"); //the SuRVoS
annotation is made binary
run("Morphological Filters (3D)", "operation=Closing element=Cube x-radius=" + radiusValue + "
y-radius=" + radiusValue + " z-radius=" + radiusValue); //smooths edges of inclusion
if (runFillholes == true) {
    run("Fill Holes", "stack"); //fill holes in annotation if ticked checkbox at start
}
//not needed for 11 but helps 1

imageCalculator("AND create stack", filename, annotation); //combines the binary data and
annotation to black-out the cytosol

selectWindow("Result of " + filename);
run("Fill Holes", "stack"); //fills gaps within bacteria cells

run("Morphological Filters (3D)", "operation=Erosion element=Ball x-radius=" + radiusValue + "
y-radius=" + radiusValue + " z-radius=" + radiusValue);
run("Morphological Filters (3D)", "operation=Dilation element=Ball x-radius=" + radiusValue + "
y-radius=" + radiusValue + " z-radius=" + radiusValue);
//erode away speckle/bumps and edge of bacteria but then dilate back to full volume of bacteria
without bringing the speckle/bumps back
    // this will be completed using the radius chosen at the start

run("Distance Transform Watershed 3D", "distances=[Borgefors (3,4,5)] output=[16 bits]
normalize dynamic=" + dynamicValue + " connectivity=6");
//separates touching objects

run("Morphological Filters (3D)", "operation=Erosion element=Ball x-radius=" + radiusValue + "
y-radius=" + radiusValue + " z-radius=" + radiusValue);
//makes the separations thicker such that touching objects are not counted as one object
Dialog.addNumber("Choose morphological radius", 1);
run("8-bit");

selectImage("Result-Erosion-Dilationdist-watershed-Erosion");
z=nSlices;
for (i=0; i>z; i++ ) {
```

```
   run("Analyze Particles...", "  circularity=" + circularityMin + "-" + circularityMax + "
show=Masks display stack");
   run("Invert LUT");
}
//removes non-circular obejcts e.g. background speckle that otherwise contributes to volume of
bacteria
   // this will be completed using the circularity range chosen at the start

if (runDespeckle == true) {
   run("Despeckle", "stack"); //final despeckle if ticked checkbox at start
}

run("3D Objects Counter", "threshold=1 slice=76 size=" + sizeMin + "-" + sizeMax + " objects
surfaces statistics summary");
//default min.=250 max.=50000
//counts objects, gives statistics window with volumes and objects map with numbered objects

//A window will open for you to select your raw tomogram for merge with the numbered surface
map
filepath = File.openDialog("Reselect tomogram for Merge");
open(filepath);
filename = File.getName(filepath);

run("Merge Channels...", "c1=[Surface map of Result-Erosion-Dilationdist-watershed-Erosion]
c4=data-1.tif");
//c4=data-1.tif is universal if using the raw exported data from SuRVoS as specified at start
//observe outlined and numbered bacteria
```
